# Investigating Genomic Differences by Ethnicity in Breast, Colorectal and Prostate Cancers: Secondary Data Analysis of the Genomic Data Commons (GDC) Database

**DOI:** 10.1002/cam4.71018

**Published:** 2025-10-24

**Authors:** Jack Carr, Mark A. Faghy, David Broom, Ruth E. M. Ashton

**Affiliations:** ^1^ Research Centre for Physical Activity, Sport and Exercise Sciences (PASES), Coventry University Coventry UK; ^2^ Biomedical and Clinical Exercise Science Research Theme University of Derby Derby UK

**Keywords:** cancer, epidemiology, ethnicity

## Abstract

**Objective:**

Globally, millions of cancer cases are diagnosed annually and mortality rates continue to rise with breast (BC), colorectal (CRC) and prostate (PC) cancer among the most prevalent. Race and ethnic disparities in cancer outcomes have been well‐documented; however, the underlying factors contributing to these disparities are currently unknown.

**Design:**

This study utilised the Genomic Data Commons (GDC) Portal, a publicly accessible repository, therefore ethical approval was not required. Cancer incidence data were collected by prevalent gene mutations associated with BC, CRC and PC within White, Black and Asian populations. Rolling one‐year survival rates were constructed for each genetic mutation.

**Results:**

For BC, Black and Asian individuals exhibited higher percentages of cases associated with TP53 mutations compared to Whites. CRC incidence showed Black individuals exhibited higher percentages of cases associated with APC, KRAS and PIK3CA mutations compared to Whites and Asians. PC incidence demonstrated that Black individuals had elevated percentages of cases associated with SPOP, ATM and SYNE1 mutations compared to Whites and Asians. Asian individuals displayed significantly lower survival percentages over 10 years compared to White and Black populations across genetic mutations associated with BC. White individuals exhibited significantly higher survival percentages over 10 years compared to Black individuals across genetic mutations associated with CRC.

**Conclusion:**

Significant disparities exist in cancer incidence and survival rates across White, Black and Asian populations. These findings demonstrate the importance of targeted approaches in cancer prevention, diagnosis and treatment to address disparities and the need for equitable healthcare. Further research is needed to identify mechanisms driving such disparities and to develop effective strategies to improve cancer outcomes across diverse ethnic populations.

## 1 Introduction

Cancer is a formidable challenge to public health globally and is recognised as the second most prevalent cause of mortality in the USA [1]. As recently as 2022, there were an estimated 20 million new cancer cases and 9.7 million deaths annually worldwide [2]. Breast (BC), colorectal (CRC) and prostate (PC) cancers collectively constituted 28% of all cancer diagnoses in 2023, exerting considerable strain on public health resources across the globe [3]. CRC and PC accounted for 24% of cancer diagnoses and 16% of cancer‐related deaths among men in 2022, while BC and CRC contributed to 32% of cancer diagnoses and 25% of cancer‐related deaths among women in the same year [4]. Furthermore, CRC emerged as the leading cause of mortality among males aged 20 to 49, while BC held this distinction among females within the same age group in 2020 [2].

Health disparities in cancer diagnosis and care have been well‐documented, with significant variations observed among different racial and ethnic groups [3]. For instance, studies have consistently highlighted disparities in BC incidence and mortality rates, with Black women experiencing higher incidence rates and poorer outcomes compared to their White counterparts [5]. The American Cancer Society's (ACS) Cancer statistics (2023) suggest that incidence rates of BC are 5% higher among White people compared to Black people but mortality rates are 40% higher in Black people compared to White people. Similarly, disparities in CRC and PC incidence and mortality rates have been observed, with Black men exhibiting higher incidence rates and worse survival outcomes compared to White and Asian men [6, 7]. ACS Cancer Statistics (2023) supports this, showing a 13% higher incidence rate and 23% higher mortality rate of PC and a 31% higher incidence rate and 65% higher mortality rate of CRC among Black people compared to White and Asian people.

Evidence suggests that genomic differences among ethnicities may contribute to disparities in cancer incidence and treatment response [8]. Genetic variations have been identified across different racial and ethnic groups, influencing cancer predisposition, tumour biology and outcomes [9]. Understanding the underlying genomic mechanisms in BC, CRC and PC among different ethnicities is essential to elucidate these disparities. Genomic studies have revealed diverse patterns of genetic mutations across ethnic groups, reflecting underlying genetic diversity in tumour biology [10]. For instance, studies have identified distinct genomic subtypes of PC associated with aggressive disease, with differential prevalence among ethnic populations [11]. Similarly, despite Black women having a lower incidence of breast cancer compared to White women, they experience higher mortality rates [12]. This disparity may be associated with distinct genetic mutations, such as a higher prevalence of TP53 mutations, which potentially contribute to the aggressive behaviour of their tumours [13]. Furthermore, CRC exhibits differences in genomic alterations, including mutations in genes such as APC, TP53 and KRAS, with variations observed in mutation frequencies and distribution among ethnic populations [7, 14, 15]. These variations have been associated with poorer prognosis and higher mortality rates in Black individuals compared to White and Asian individuals, underscoring the influence of genetic mutations on mortality disparities in cancer [16, 17].

Understanding the underlying genomic mechanisms contributing to cancer incidence and mortality disparities among ethnic groups is crucial for advancing approaches to developing targeted interventions to improve cancer prevention and treatment across diverse populations. By highlighting the complex interplay between genetic, environmental and socio‐cultural factors, we can develop more effective strategies for cancer prevention, diagnosis and treatment tailored to the unique needs of individuals from different ethnic backgrounds.

## 2 Methodology

### 2.1 Study Design

The National Cancer Institute's Genomic Data Commons (GDC, https://portal.gdc.cancer.gov/) provides a centralised platform for accessing and analysing vast quantities of genomic and clinical data from diverse cancer types. This study is a cross sectional evaluation of the National Cancer Institute's GDC Data Portal, aiming to investigate the influence of genetic mutations and ethnicity on cancer incidence and survival rates.

### 2.2 Data Collection

Data collection for this study involved identifying the most prevalent gene mutations associated with BC, CRC and PC within White, Black and Asian populations from all available genomic datasets within the GDC's Data Portal (*n* = 2824 total cases). The GDC database includes data from multiple sources which could introduce selection biases across racial groups. To ensure consistency in our analysis we combined datasets by focusing on consistently reported somatic mutations across different sources. In our analysis, the Asian population was treated as a single group due to lack of further subcategorization. Within each cancer type, relevant datasets containing genomic sequencing data, clinical characteristics and patient demographics were identified. These datasets were meticulously curated to ensure data integrity and completeness. Additionally, data was stratified by ethnicity, distinguishing between White, Black and Asian populations, and further segmented by age groups according to European age structure ranges (15–24, 25–54, 55–64, 65+) [18]. While tumour grade and stage data were available within the GDCs database, there was an insufficient number of cases for our variables to allow for robust data analysis.

#### 2.2.1 Cancer Incidence

Incidence data were collated and combined into tables using Microsoft Excel, classified by cancer type.

#### 2.2.2 Survival Rates

Survival data was acquired from the GDC Data Portal through accessing the survival data that had been individually recorded within respective studies. This process involved identification and retrieval of survival data specifically associated with the genetic mutations involved in BC, CRC and PC cases within our designated population groups. Subsequently, rolling one‐year survival rates for each genetic mutation across a span of up to 10 years were calculated, contingent upon data availability (See Figures S1–S3). Survival rates were collated into tables and graphs using Microsoft Excel, divided up by cancer type.

### 2.3 Data Analysis

For the assessment of significant differences in mutation incidence between ethnicities across BC, CRC and PC, R Studios was used to perform *Z* tests [19].

#### 2.3.1 Cancer Incidence

Percentage of cases affected by each gene mutation associated with the respective cancer types across different ethnicities (White/Black, White/Asian, Black/Asian) were compared. Statistical significance was determined by a *p* value of ≤ 0.05 [20]. Given the large number of *Z* tests conducted when analysing cancer incidence across different ethnicities, Benjamini—Hochberg corrections were applied to the obtained *p* value's to control false discovery rates.

#### 2.3.2 Survival Rates

R Studios was used to generate Kaplan–Meier curves, allowing for the visualisation of survival percentage disparities between ethnic groups for each genetic mutation within BC, CRC and PC. Additionally, Mantel–Haenszel tests and pairwise log‐rank tests were conducted in R Studios to evaluate significant differences in survival probabilities across ethnicities. Statistical significance was established at a pre‐defined prior *p* value of ≤ 0.05 [20]. Since survival rates for multiple mutations across multiple ethnicities were compared in BC, Benjamini–Hochberg corrections were applied to the *p* values obtained to control false discovery rates.

## 3 Results

The following is the result of data analysis conducted on data obtained from 2824 cancer cases from the GDC's database.

### 3.1 Ethnicity and Cancer Incidence

#### 3.1.1 Breast Cancer Incidence

Our analysis highlights notable disparities in BC incidence among different ethnic groups, particularly concerning the prevalence of TP53 and PIK3CA mutations. White individuals exhibited a lower percentage of total cases of BC associated with TP53 mutations (28%) compared to Black (38%) and Asian (46%) populations. Conversely, Black (43%) and Asian (52%) individuals demonstrated a higher percentage for TP53 mutation‐related BC than their White counterparts (25%) within the 25–54 age group. Moreover, Asian individuals displayed an elevated percentage of TP53‐related BC at ages 65 and above (58%) compared to Black (36%) and White individuals (20%) (Table 1).

**TABLE 1:** A table showing incidence rates of genetic mutations associated with breast cancer among different ethnic and age groups.

		% of cases affected by age range					
Ethnicity	Mutated gene	15–24	25–54	55–64	65+	% of total cases affected	No of total cases affected
White	TP53	0%	24.68%	30.11%	20.44%	27.72%	290/1046
	PIK3CA	0%	29.00%	29.39%	36.13%	30.59%	320/1046
Black	TP53	N/A	42.86%	31.82%	36.21%	38.21%	81/212
	PIK3CA	N/A	18.10%	13.64%	32.76%	18.87%	40/212
Asian	TP53	N/A	52.38%	38.89%	58.33%	46.43%	39/84
	PIK3CA	N/A	35.71%	38.89%	16.67%	34.52%	29/84

Following the *Z* tests conducted to assess differences in the percentage incidence of various gene mutations associated with BC among different ethnicities, significant disparities were observed (See Table S1). Specifically, there was a significant difference in BC associated with TP53 mutations between White and Black individuals, with Black individuals demonstrating a significantly higher percentage incidence (*p* < 0.01), as well as between White and Asian individuals, with Asian individuals exhibiting a significantly higher percentage incidence (*p* < 0.01). Furthermore, a significant difference was identified in BC associated with PIK3CA mutations between White and Black individuals, with White individuals showing a significantly higher percentage incidence (*p* < 0.01), as well as between Black and Asian individuals, with Asian individuals displaying a significantly higher percentage incidence (*p* < 0.01).

#### 3.1.2 Colorectal Cancer Incidence

When investigating CRC incidence among different ethnicities, we found that White (74%) and Black individuals (80%) exhibited a higher percentage of CRC cases associated with APC gene mutations compared to Asian populations (61%). Despite this, Asian (100%) and Black individuals (90%) exhibit a higher percentage of CRC cases associated with APC mutations for the 55–64 age group compared to White populations (68%). We also found that there was not much difference between percentage of total CRC cases associated with TP53 and MUC16 mutations between White (60% and 32%), Black (63% and 29%), and Asian individuals (58% and 36%).

Furthermore, the percentage of total CRC cases associated with KRAS genetic mutations was higher in Black individuals (50%) compared to White (39%) and Asian populations (30%). In CRC cases associated with OBSCN mutations, Asian people exhibited a higher percentage of total cases affected (39%) compared to White (26%) and Black people (27%) (Table 2).

**TABLE 2:** A table showing incidence rates of genetic mutations associated with colorectal cancer among different ethnic and age groups.

		% of cases affected by age range					
Ethnicity	Mutated gene	15–24	25–54	55–64	65+	% of total cases affected	No of total cases affected
White	APC	N/A	68.76%	67.65%	75.32%	73.64%	271/368
	TP53	N/A	64.06%	58.82%	62.66%	59.78%	220/368
	KRAS	N/A	35.94%	38.24%	43.67%	39.40%	145/368
	MUC16	N/A	31.25%	26.47%	34.18%	31.79%	117/368
	PIK3CA	N/A	21.88%	22.06%	24.68%	22.83%	84/368
	TTN	N/A	51.56%	50%	65.82%	58.42%	215/368
	SYNE1	N/A	31.25%	23.53%	36.71%	32.34%	119/368
	OBSCN	N/A	23.44%	25%	28.48%	26.90%	99/368
Black	APC	N/A	70%	90%	78.57%	80.26%	61/76
	TP53	N/A	50%	65%	64.29%	63.16%	48/76
	KRAS	N/A	45%	30%	67.86%	50%	38/76
	MUC16	N/A	25%	20%	35.71%	28.95%	22/76
	PIK3CA	N/A	0%	50%	42.86%	30.26%	23/76
	TTN	N/A	45%	45%	42.86%	44.74%	34/76
	SYNE1	N/A	15%	30%	42.86%	27.63%	21/76
	OBSCN	N/A	20%	40%	17.86%	26.32%	20/76
Asian	APC	N/A	33.33%	100%	20%	60.61%	20/33
	TP53	N/A	33.33%	80%	80%	57.58%	19/33
	KRAS	N/A	50%	20%	20%	30.30%	10/33
	MUC16	N/A	33.33%	20%	40%	36.36%	12/33
	PIK3CA	N/A	33.33%	20%	0%	21.21%	7/33
	TTN	N/A	50%	60%	20%	57.58%	19/33
	SYNE1	N/A	16.67%	40%	40%	33.33%	11/33
	OBSCN	N/A	33.33%	40%	40%	39.39%	13/33

Following the conduction of *Z* tests assessing the difference in the percentage incidence of various gene mutations associated with CRC, significant disparities emerged across several genetic mutations (See Table S3). Notably, the *Z* tests showed significant difference in CRC associated with APC, KRAS and TNN mutations between Black and Asian individuals with Black populations having a higher percentage incidence across all of these mutations (*p* < 0.05). However, after the application of the Benjamini—Hochberg corrections, these differences were no longer statistically significant. This suggests that while the differences were very close to being significant, the correction for multiple testing adjusted the *p* values above the significance threshold.

#### 3.1.3 Prostate Cancer Incidence

Our analysis also found that White individuals exhibited a slightly higher percentage of PC cases associated with TP53 mutations (11%) compared to Black (6%) and Asian populations (6%). Interestingly, within the 25–54 age group, Asian individuals exhibited the highest percentage of PC cases associated with TP53 mutations (20%), followed by White individuals (16%) and then Black individuals (6%). Notably, a significantly higher percentage of PC cases associated with FOXA1 mutations were observed in Asian individuals in the 55–64 and 65+ age groups (25% and 14%) compared to White (5% and 7%) and Black populations of the same age groups (7% and 12%). Additionally, in PC associated with LRP1B mutations, a significantly higher percentage of total PC cases were observed in Asian individuals in the 25–54 age group (40%) compared to White (3%) and Black individuals (0%) of the same age group. Finally, a significantly higher percentage of total PC cases associated with HECTD4 mutations were observed in Asian individuals (19%) compared to White individuals (1%), with no cases observed among Black individuals (Table 3).

**TABLE 3:** A table showing incidence rates of genetic mutations associated with prostate cancer among different ethnic and age groups.

		% of affected cases by age range					
Ethnicity	Mutated gene	15–24	25–54	55–64	65+	% of total cases affected	No of total cases affected
White	TP53	N/A	16.44%	11.16%	10.48%	11.41%	64/561
	SPOP	N/A	5.48%	9.56	8.73%	8.56%	48/561
	ATM	N/A	1.37%	2.39%	3.93%	2.85%	16/561
	KMT2D	N/A	1.37%	5.98%	5.68%	5.17%	29/561
	FOXA1	N/A	4.11%	5.18%	7.42%	5.88%	33/561
	MUC16	N/A	6.85%	6.37%	4.37%	5.53%	31/561
	LRP1B	N/A	2.74%	2.79%	5.24%	3.74%	21/561
	TNN	N/A	4.11%	11.95%	8.73%	9.63%	54/561
	SYNE1	N/A	2.74%	3.98%	4.37%	4.10%	23/561
	HECTD4	N/A	0%	0.40%	0.87%	0.53%	3/561
Black	TP53	N/A	6.25%	6.90%	5.88%	6.35%	4/63
	SPOP	N/A	6.25%	10.34%	23.53%	14.29%	9/63
	ATM	N/A	6.25%	13.79%	0%	7.94%	5/63
	KMT2D	N/A	0%	10.34%	5.88%	6.35%	4/63
	FOXA1	N/A	0%	6.90%	11.76%	6.35%	4/63
	MUC16	N/A	0%	10.34%	11.76%	7.94%	5/63
	LRP1B	N/A	0%	6.90%	5.88%	4.76%	3/63
	TNN	N/A	6.25%	10.34%	11.76%	9.52%	6/63
	SYNE1	N/A	0%	6.90%	23.53%	9.52%	6/63
	HECTD4	N/A	0%	0%	0%	0%	0/63
Asian	TP53	N/A	20%	0%	0%	6.25%	1/16
	SPOP	N/A	0%	25%	0%	6.25%	1/16
	ATM	N/A	0%	0%	0%	0%	0/16
	KMT2D	N/A	0%	0%	0%	0%	0/16
	FOXA1	N/A	0%	25%	14.29%	12.50%	2/16
	MUC16	N/A	0%	0%	0%	0%	0/16
	LRP1B	N/A	40%	0%	0%	12.50%	2/16
	TNN	N/A	0%	0%	0%	0%	0/16
	SYNE1	N/A	0%	0%	0%	0%	0/16
	HECTD4	N/A	40%	25%	0%	18.75%	3/16

Following the conduction of Z‐tests assessing differences in the percentage incidence of various gene mutations associated with PC, significant disparities emerged across several genetic mutations (See Table S2). Notably, there was a significant difference in PC associated with ATM mutations between White and Asian individuals, with White individuals demonstrating a significantly higher percentage incidence (*p* < 0.01), and between Black and Asian individuals, with Black individuals exhibiting a significantly higher percentage incidence (*p* < 0.05). Similarly, significant differences were observed in PC associated with KMT2D gene mutations between White and Asian individuals, with White individuals having a significantly higher percentage incidence (*p* < 0.01). Furthermore, significant differences were noted in PC associated with MUC16 mutations between White and Asian individuals, with White individuals exhibiting a higher percentage incidence (*p* < 0.01), and between Black and Asian individuals, with Black individuals having a higher percentage incidence (*p* < 0.05). Additionally, significant differences were observed in PC associated with TNN gene mutations between White and Asian individuals, with White individuals showing a higher percentage incidence (*p* < 0.01), and between Black and Asian individuals, with Black individuals demonstrating a significantly higher percentage incidence (*p* = 0.01). Lastly, significant differences were noted in PC associated with SYNE1 mutations between White and Asian individuals, with White individuals having a significantly higher percentage incidence (*p* < 0.01), and between Black and Asian individuals, with Black individuals exhibiting a higher percentage incidence (*p* < 0.05).

### 3.2 Ethnicity and Cancer Survival Rate

#### 3.2.1 Breast Cancer Survival Rate

After calculating the rolling 10‐year survival rates for BC in our different ethnic populations for TP53 and PIK3CA mutations, we created Kaplan–Meier curves and performed Pairwise Log‐Rank tests to test for significant differences between the survival rates of different ethnicities. We found that there were significant differences between the survival rates of Black and Asian people (*p* < 0.01, CI: 1.21–2.61), as well as White and Asian people (CI: 1.59–3.04) for BC associated with both TP53 and PIK3CA mutations. We did not find a significant difference between White and Black people for either mutation. The data collected suggest that Asian individuals have a significantly reduced survival probability for BC associated with TP53 and PIK3CA mutations compared to White and Black people.

#### 3.2.2 Colorectal Cancer Survival Rate

**FIGURE 1:**
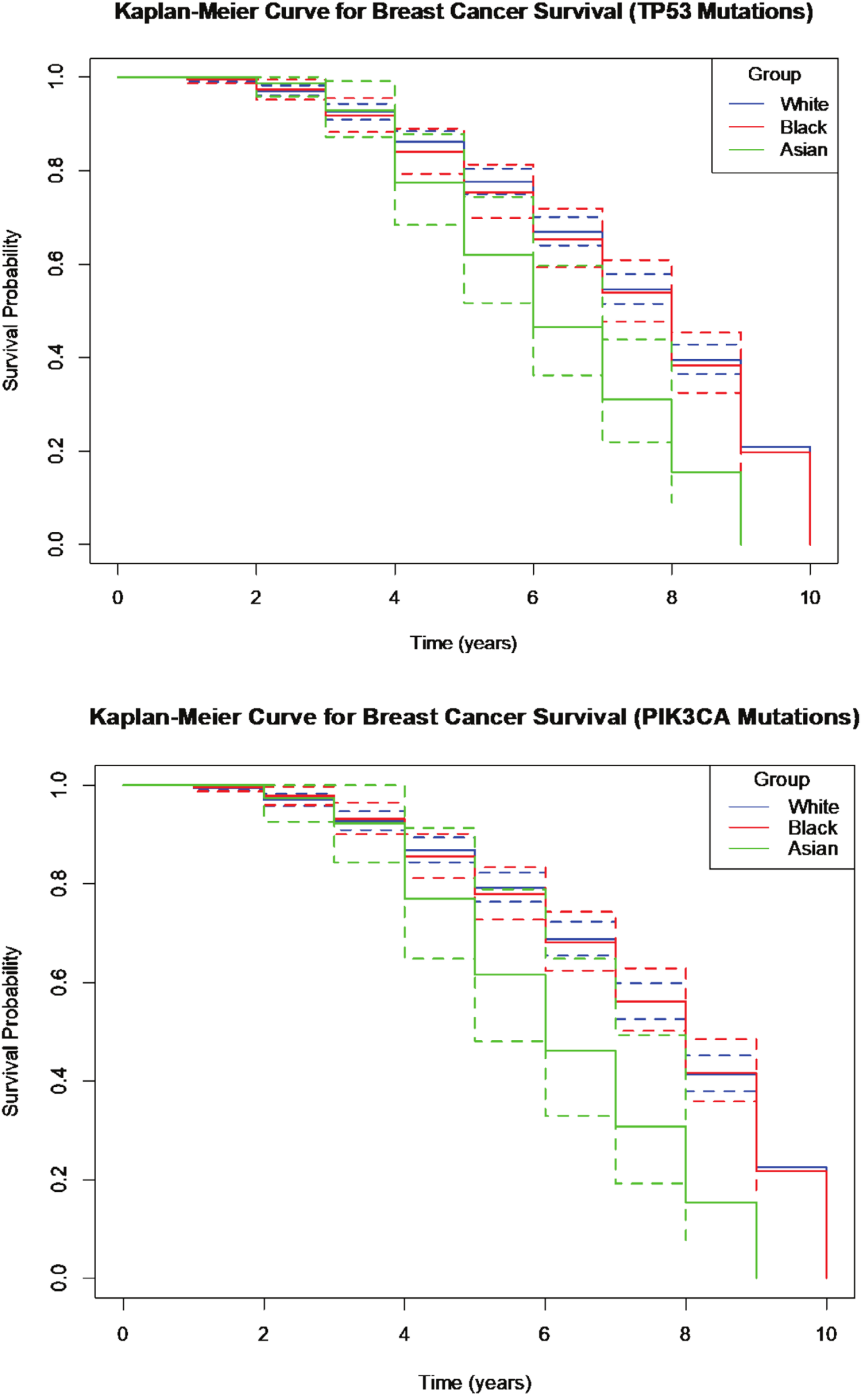
Panel plots of Kaplan–Meier curves showing survival probability for breast cancer associated with different genetic mutations between White, Black and Asian.

**FIGURE 2:**
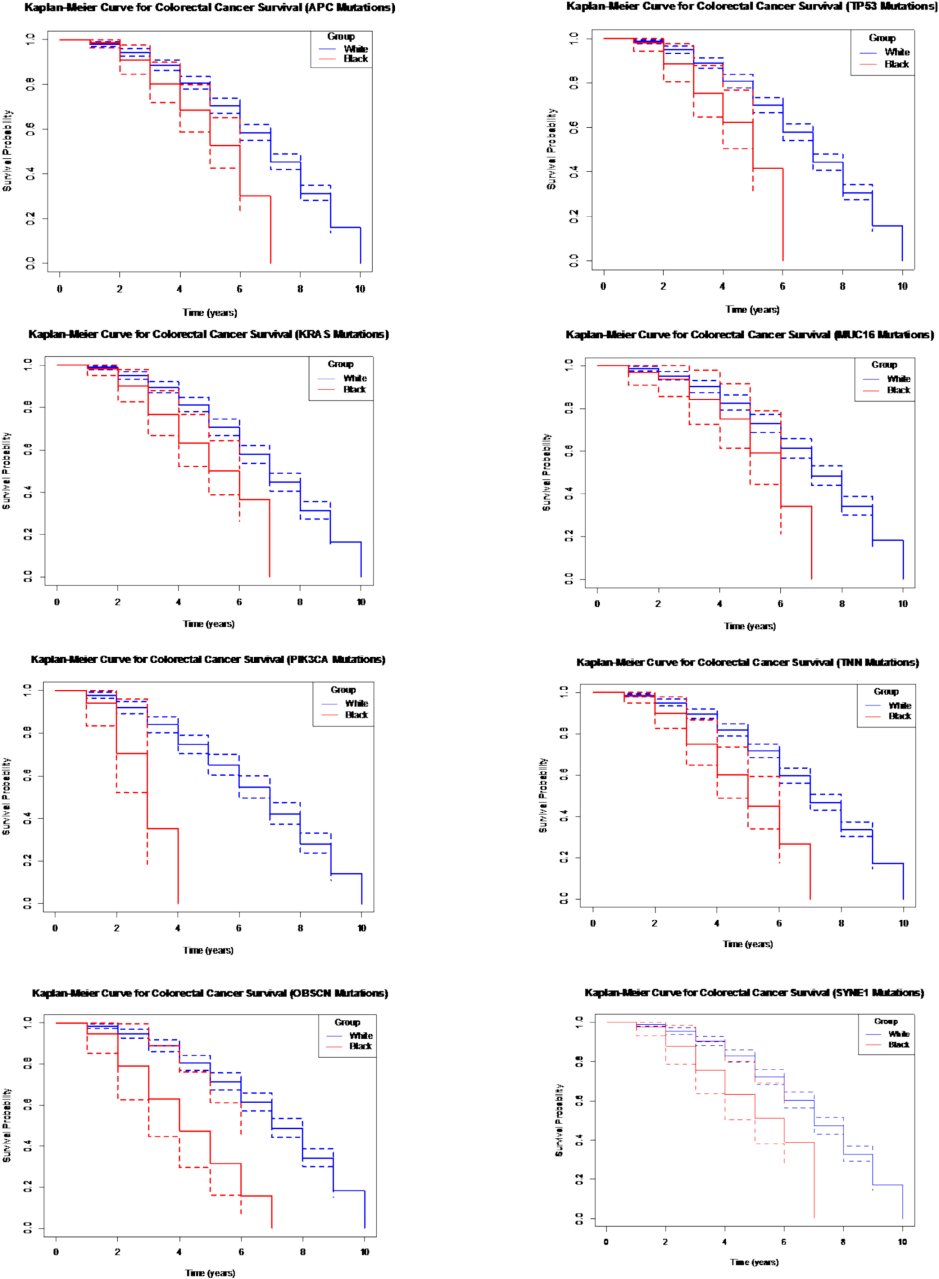
Panel plots of Kaplan–Meier curves showing survival probability for colorectal cancers associated with different genetic mutations between white and black individuals.

We were able to create rolling 10‐year survival probability and Kaplan–Meier curves for CRC in White and Black populations; however, there was not enough survival data recorded in Asian populations with CRC. Therefore, we performed Pairwise Log‐Rank tests exploring the difference in survival probability between White and Black people for all the different genetic mutations most associated with CRC development. The data suggests that there was a significant difference in survival probability between White and Black populations in all genetic mutations we explored (*p* < 0.01). This suggests that White individuals have a significantly higher survival probability compared to Black individuals diagnosed with CRC associated with any of the most common genetic mutations related to CRC development (Figures 1 and 2).

#### 3.2.3 Prostate Cancer Survival Rate

**FIGURE 3:**
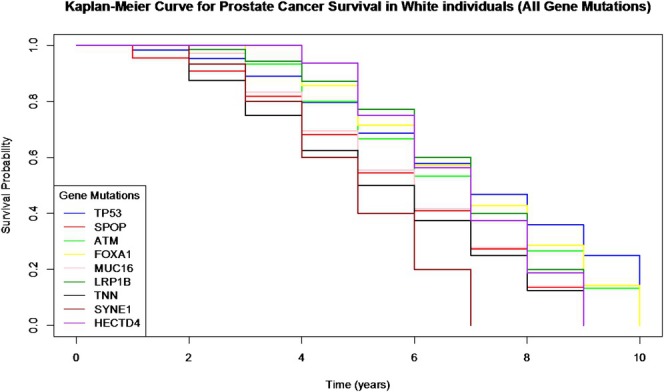
Kaplan–Meier curves showing survival probability for prostate cancer associated with different genetic mutations between White individuals only.

There was only enough survival data to calculate rolling 10‐year survival probability and Kaplan–Meier curves for PC in White populations as there was not enough survival data recorded in Black or Asian populations with PC. Therefore, we used the data collected to create a Kaplan–Meier curve exploring the survival probability between the different genetic mutations most associated with PC cases in White individuals. After the conduction of Pairwise Log‐Rank tests, we found that there was a significant difference in survival probability between PC associated with TP53 mutations and SYNE1 mutations (*p* < 0.05: CI: 0.93–3.49), FOXA1 mutations and SYNE1 mutations (*p* < 0.05, CI: 0.82–1.72), and LRP1B mutations and SYNE1 mutations (*p* < 0.01, CI: 1.65–7.14). This data suggests that White people diagnosed with PC due to SYNE1 mutations have a significantly reduced survival probability compared to White people diagnosed with PC due to TP53, FOXA1 and LRP1B mutations (Figure 3).

## 4 Discussion

Our secondary data analysis highlights novel insights into the relationship between genetic mutations, ethnicity and cancer outcomes. Through a comprehensive analysis of data obtained from the GDC Data Portal, we investigated the incidence rates and survival outcomes of BC, CRC and PC across different ethnic groups. Our findings for the first time highlight substantial disparities in cancer incidence and survival rates among White, Black and Asian populations, highlighting the influence of genetic mutations on cancer outcomes.

Our results demonstrate a difference in the incidence rates of various gene mutations associated with BC, CRC and PC among different ethnicities. For BC, TP53 mutations were significantly more prevalent in Black and Asian populations compared to White individuals. This aligns with previous research indicating a higher frequency of aggressive BC subtypes, such as triple‐negative breast cancer, which are often driven by TP53 mutations, in Black women [13, 21, 22, 23]. It is important to acknowledge that BC is known to be a heterogeneous disease, with variations in tumour types based on hormonal status such as ER, PR and HER2 (Iqbal and Buch, 2016). However, in our analysis, hormonal status on these tumour subtypes was unavailable in the GDCs database. As a result, our data identifies racial differences in genetic mutations but cannot factor in the influence of racial differences in hormonal status.

In PC, we observed a difference in the prevalence of several gene mutations, including ATM, KMT2D, MUC16, TNN and SYNE1, among White, Black and Asian populations. These disparities could be linked to the underlying genetic diversity and the influence of ancestry on cancer susceptibility. For instance, the higher incidence of ATM mutations in White individuals could be related to inherited mutations that predispose them to PC [24], while the higher prevalence of KMT2D and MUC16 mutations in Black individuals may contribute to the aggressive nature of PC observed in this population [25, 26, 27].

Colorectal incidence also exhibited significant variations in gene mutations across ethnicities. The higher percentage of CRC cases associated with APC and KRAS mutations in Black individuals compared to Asian populations is consistent with previous findings that suggest distinct mutational landscapes in CRC among different racial groups [7, 15, 28]. These genetic differences may partly explain the poorer prognosis and higher mortality rates observed in Black individuals with CRC [29, 30].

The survival analysis revealed that ethnic disparities extend beyond cancer incidence to impact survival outcomes. Our Kaplan–Meier curves and Pairwise Log‐Rank tests indicated that Asian individuals had a significantly reduced survival probability for BC associated with TP53 and PIK3CA mutations compared to White and Black individuals. This may be due to a combination of factors, including later‐stage diagnosis [31], differences in access to healthcare [32] and potential biological differences in tumour behaviour [33].

A lack of sufficient survival data for Black and Asian populations in PC limited our analysis to only White individuals. However, the significant differences in survival probability between different genetic mutations in White individuals underscore the importance of understanding the genetic drivers of cancer to inform targeted therapies. For instance, the reduced survival probability associated with SYNE1 mutations highlights the need for further research into the biological mechanisms underlying these mutations and their impact on treatment response [34]. The lack of survival data for Black and Asian populations also highlights the crucial need to address the gaps in survival data for ethnic minorities for healthcare equity and equality [35]. Without comprehensive data, it is challenging to develop effective treatment strategies and interventions that are inclusive of all populations. Efforts to enhance data collection and reporting for underrepresented groups are essential to ensure that all individuals benefit from advancements in cancer research and treatment [36].

Colorectal survival analysis showed Black individuals had a significantly lower survival probability compared to White individuals, suggesting that genetic factors, combined with socio‐economic and healthcare access disparities, contribute to these outcomes [7, 37]. The higher incidence of aggressive mutations, such as KRAS, in Black individuals may also play a role in the observed survival differences [38]. Addressing these disparities requires a multifaceted approach that includes improving access to early detection and treatment, as well as targeted research into the genetic underpinnings of CRC in diverse populations [30].

The disparities observed in this study are multifaceted and likely result from a combination of genetic, environmental and socio‐economic factors. Genetic predispositions, such as the prevalence of certain mutations in specific ethnic groups, can influence cancer susceptibility and progression [9, 17]. Environmental factors, including lifestyle, diet and exposure to carcinogens, may further contribute to these disparities [39]. Additionally, socio‐economic factors, such as access to healthcare, quality of medical care and cultural attitudes towards health, can significantly impact cancer diagnosis, treatment and outcomes [40, 41].

To address these disparities, it is essential to adopt a multi‐pronged approach that includes improving access to healthcare, promoting early detection and screening programmes and developing tailored therapies based on the genetic profiles of diverse populations [30]. Further research is needed to elucidate the biological mechanisms underlying the observed genetic differences and to explore how these differences interact with environmental and socio‐economic factors to influence cancer outcomes [40, 42].

The strengths of this study are its comprehensive analysis of a large dataset from the GDC, allowing for robust statistical comparisons across different ethnic groups. Additionally, the study provides valuable insights into the genetic underpinnings of cancer disparities, which can inform targeted therapeutic strategies and public health interventions. However, there are limitations to consider, including the lack of survival data for Asian and Black patients living with prostate cancer. The limited data availability may hinder the generalizability of the findings and underscores the need for more comprehensive data collection in diverse populations. The potential for selection bias due to the nature of the dataset, which may not be fully representative of the broader population. Additionally, the study's retrospective design limits the ability to establish causality, and the lack of detailed environmental, lifestyle and more detailed ethnic subgroup data makes it difficult to fully account for these factors in the observed disparities.

Healthcare professionals and practitioners should use these findings to develop more personalised approaches to cancer screening, diagnosis and treatment that consider the genetic profiles and unique risk factors of diverse ethnic groups. Furthermore, these findings underscore the need for improved access to genetic testing and counselling services, particularly for underrepresented populations, to better address and mitigate cancer disparities. Additionally, our findings emphasise the importance of developing targeted screening and prevention strategies tailored to high‐risk populations. Efforts should be made by policy‐makers to address disparities in access to early detection and treatment, particularly in underrepresented groups, through community outreach and culturally sensitive healthcare interventions.

Finally, the lack of tumour stage and grade data prevented us from fully accounting for differences in disease severity at diagnosis. As a result, the observed survival disparities between ethnic groups and genetic mutations may be partially influenced by variations in stage at presentation rather than genetic factors alone. Future studies incorporating stage and grade‐specific analyses are needed to better isolate the impact of genetic mutations on survival outcomes in diverse ethnic populations is warranted.

In conclusion, our study highlights the significant impact of genetic mutations on cancer incidence and survival across different ethnic groups. By understanding the genetic underpinnings of cancer disparities, we can develop more effective strategies for prevention, diagnosis and treatment, ultimately improving cancer outcomes for all individuals, regardless of their ethnic background [7, 43].

## Author Contributions

**Jack Carr:** investigation, writing – original draft, methodology, validation, visualization, writing – review and editing, software, formal analysis, data curation, conceptualization. **Mark A. Faghy:** conceptualization, writing – review and editing, visualization, validation, project administration, supervision, resources. **David Broom:** writing – review and editing, visualization, validation, project administration, supervision, resources. **Ruth E. M. Ashton:** writing – original draft, conceptualization, validation, visualization, writing – review and editing, methodology, project administration, supervision, resources.

## Disclosure

Jack Carr is a PhD student at Coventry University, and this research was conducted as part of their doctoral studies.

## Conflicts of Interest

The authors declare no conflicts of interest.

## Supporting information


Data S1.

## Data Availability

The data that support the findings of this study are openly available in Genomic Data Commons Data Portal at https://portal.gdc.cancer.gov/.
