# Supplementary material for: Investigating Genomic Differences by Ethnicity in Breast, Colorectal and Prostate Cancers: Secondary Data Analysis of the Genomic Data Commons (GDC) Database
Source: Cancer Med. 2025 Oct 24;14(20):e71018. doi: 10.1002/cam4.71018 (PMC12551590; doi:10.1002/cam4.71018)
Supplement: Supplementary file 1 — Data S1. [file CAM4-14-e71018-s001.docx]

**Supplementary material GDC Study**

**Figure S1.** Line graph showing the yearly rolling survival percentage of gene mutations causing breast cancer among different ethnic groups for up to 10 years.

**Figure S2.** Line graph showing the yearly rolling survival percentage of gene mutations causing prostate cancer among white individuals for up to 10 years.

**Figure S3.** Line graph showing the yearly rolling survival percentage of gene mutations causing colorectal cancer among white and black individuals for up to 10 years.

**Table S1.** A table showing the results of Z-Tests investigating the difference between incidence rates of genetic mutations causing breast cancer between different ethnicities.

| **Gene Mutation** | **TP53** |  |  | **PIK3CA** |  |  |
| --- | --- | --- | --- | --- | --- | --- |
| **Ethnicities compared** | White/ Black | White/ Asian | Black/ Asian | White/ Black | White/ Asian | Black/ Asian |
| **Z – Statistic** | -2.903572 | -3.332281 | -1.287728 | 3.85326 | -0.7305509 | -2.678817 |
| **P – Value** | 0.0036893 | 0.000861372 | 0.1978405 | 0.000116556 | 0.4650535 | 0.00738828 |
| **BH-adjusted significance threshold** | 0.03333 | 0.01667 | 0.05 | 0.01667 | 0.05 | 0.03333 |

**Table S2.** A table showing the results of Z-Tests investigating the difference between incidence rates of genetic mutations causing prostate cancer between different ethnicities.

| **Gene Mutation** | **TP53** |  |  | **SPOP** |  |  |
| --- | --- | --- | --- | --- | --- | --- |
| **Ethnicities compared** | White/ Black | White/ Asian | Black/ Asian | White/ Black | White/ Asian | Black/ Asian |
| **Z - Statistic** | 1.509196 | 0.8324433 | 0.01473452 | -1.255287 | 0.374651 | 1.073793 |
| **P – Value** | 0.1312488 | 0.4051588 | 0.988244 | 0.2093748 | 0.70792 | 0.2829154 |
| **BH-adjusted significance threshold** | 0.01667 | 0.03333 | 0.05 | 0.01667 | 0.03333 | 0.05 |
| **Gene Mutation** | **ATM** |  |  | **KMT2D** |  |  |
| **Ethnicities compared** | White/ Black | White/ Asian | Black/ Asian | White/ Black | White/ Asian | Black/ Asian |
| **Z - Statistic** | -1.463513 | 4.056789 | 2.331015 | -0.3674382 | 5.530365 | 2.066823 |
| **P – Value** | 0.1433272 | 4.975191 e-05 | 0.01975258 | 0.7132922 | 3.195644 e-08 | 0.03875085 |
| **BH-adjusted significance threshold** | 0.01667 | 0.03333 | 0.05 | 0.05 | 0.01667 | 0.03333 |
| **Gene Mutation** | **FOXA1** |  |  | **MUC16** |  |  |
| **Ethnicities compared** | White/ Black | White/ Asian | Black/ Asian | White/ Black | White/ Asian | Black/ Asian |
| **Z - Statistic** | -0.1455602 | -0.7949644 | -0.6972507 | -0.7381624 | 5.620033 | 2.331015 |
| **P – Value** | 0.8842686 | 0.4266342 | 0.4856459 | 0.4604157 | 1.909213 e-08 | 0.01975258 |
| **BH-adjusted significance threshold** | 0.05 | 0.01667 | 0.03333 | 0.05 | 0.01667 | 0.03333 |
| **Gene Mutation** | **LRP1B** |  |  | **TNN** |  |  |
| **Ethnicities compared** | White/ Black | White/ Asian | Black/ Asian | White/ Black | White/ Asian | Black/ Asian |
| **Z - Statistic** | -0.3643405 | -1.054572 | -0.8904479 | 0.02563061 | 7.727389 | 2.574616 |
| **P – Value** | 0.7156034 | 0.2916213 | 0.3732254 | 0.979552 | 1.097745 e-14 | 0.01003514 |
| **BH-adjusted significance threshold** | 0.05 | 0.01667 | 0.03333 | 0.05 | 0.01667 | 0.03333 |
| **Gene Mutation** | **SYNE1** |  |  | **HECTD4** |  |  |
| **Ethnicities compared** | White/ Black | White/ Asian | Black/ Asian | White/ Black | White/ Asian | Black/ Asian |
| **Z - Statistic** | -1.429616 | 4.897383 | 2.574616 | 1.728914 | -1.866302 | -1.921538 |
| **P – Value** | 0.1528272 | 9.71215 e-07 | 0.01003514 | 0.08382455 | 0.06199918 | 0.05466394 |
| **BH-adjusted significance threshold** | 0.05 | 0.01667 | 0.03333 | 0.05 | 0.03333 | 0.1667 |

**Table S3.** A table showing the results of Z-Tests investigating the difference between incidence rates of genetic mutations causing colorectal cancer between different ethnicities.

| **Gene Mutation** | **APC** |  |  | **TP53** |  |  |
| --- | --- | --- | --- | --- | --- | --- |
| **Ethnicities compared** | White/ Black | White/ Asian | Black/ Asian | White/ Black | White/ Asian | Black/ Asian |
| **Z - Statistic** | -1.29527 | 1.478953 | 2.035501 | -0.554549 | 0.2451262 | 0.5455078 |
| **P – Value** | 0.1952272 | 0.1391529 | 0.04180044 | 0.5792031 | 0.8003587 | 0.5854043 |
| **BH-adjusted significance threshold** | 0.05 | 0.03333 | 0.01667 | 0.01667 | 0.05 | 0.03333 |
| **Gene Mutation** | **KRAS** |  |  | **MUC16** |  |  |
| **Ethnicities compared** | White/ Black | White/ Asian | Black/ Asian | White/ Black | White/ Asian | Black/ Asian |
| **Z - Statistic** | -1.689087 | 1.083906 | 2.001346 | 0.4947043 | -0.5241733 | -0.7516584 |
| **P – Value** | 0.09120271 | 0.2784064 | 0.04535508 | 0.6208089 | 0.600158 | 0.4522565 |
| **BH-adjusted significance threshold** | 0.03333 | 0.05 | 0.01667 | 0.05 | 0.03333 | 0.1667 |
| **Gene Mutation** | **PIK3CA** |  |  | **TTN** |  |  |
| **Ethnicities compared** | White/ Black | White/ Asian | Black/ Asian | White/ Black | White/ Asian | Black/ Asian |
| **Z - Statistic** | -1.302207 | 0.2175958 | 1.022041 | 2.186869 | 0.0935546 | -1.243925 |
| **P – Value** | 0.1928457 | 0.827744 | 0.3067615 | 0.02875209 | 0.925463 | 0.2135273 |
| **BH-adjusted significance threshold** | 0.1667 | 0.05 | 0.03333 | 0.1667 | 0.05 | 0.03333 |
| **Gene Mutation** | **SYNE1** |  |  | **OBSCN** |  |  |
| **Ethnicities compared** | White/ Black | White/ Asian | Black/ Asian | White/ Black | White/ Asian | Black/ Asian |
| **Z - Statistic** | 0.8293038 | -0.115647 | -0.5890171 | 0.1044071 | -1.417035 | -1.321194 |
| **P – Value** | 0.4069325 | 0.9079323 | 0.5558498 | 0.9168463 | 0.1564727 | 0.1864366 |
| **BH-adjusted significance threshold** | 0.1667 | 0.05 | 0.03333 | 0.05 | 0.01667 | 0.03333 |

**Figure S4.** A list of every genetic mutation studied in this paper.

**Breast Cancer (BC) mutations studied:**

1. TP53
2. PIK3CA

**Colorectal Cancer (CRC) mutations studied:**

1. APC
2. TP53
3. KRAS
4. MUC16
5. PIK3CA
6. TTN
7. SYNE1
8. OBSCN

**Prostate Cancer (PC) mutations studied:**

1. TP53
2. SPOP
3. ATM
4. KMT2D
5. FOXA1
6. MUC16
7. LRP1B
8. TNN
9. SYNE1
10. HECTD4
